# Hemin Prevents Increased Glycolysis in Macrophages upon Activation: Protection by Microbiota-Derived Metabolites of Polyphenols

**DOI:** 10.3390/antiox9111109

**Published:** 2020-11-11

**Authors:** Catalina Carrasco-Pozo, Kah Ni Tan, Vicky M. Avery

**Affiliations:** 1 Discovery Biology, Griffith Institute for Drug Discovery, Griffith University, Nathan, Brisbane 4111, Queensland, Australia; kahni.tan@griffith.edu.au (K.N.T.); v.avery@griffith.edu.au (V.M.A.); 2 CRC for Cancer Therapeutics, Griffith University, Nathan, Brisbane 4111, Queensland, Australia

**Keywords:** macrophage, hemin, 3,4-dihydroxyphenylacetic acid, 4-hydroxyphenylacetic acid, glycolysis, mitochondria

## Abstract

Meat consumption plays a critical role in the development of several types of cancer. Hemin, a metabolite of myoglobin produced after meat intake, has been demonstrated to be involved in the cancer initiation phase. Macrophages are key components of the innate immunity, which, upon activation, can prevent cancer development by eliminating neoplastic cells. Metabolic reprogramming, characterized by high glycolysis and low oxidative phosphorylation, is critical for macrophage activation. 3,4-dihydroxyphenylacetic acid (3,4DHPAA) and 4-hydroxyphenylacetic acid (4HPAA), both microbiota-derived metabolites of flavonoids, have not been extensively studied although they exert antioxidant properties. The aim of this study was to determine the effect of hemin on the anticancer properties of macrophages and the role of 3,4DHPAA and 4HPAA in metabolic reprogramming and activation of macrophages leading to the elimination of cancer cells. The results showed that hemin inhibited glycolysis, glycolytic, and pentose phosphate pathway (PPP) enzyme activities and hypoxia-inducible factor-1 alpha (HIF-1α) stabilization, which interferes with macrophage activation (evidenced by decreased interferon-γ-inducible protein 10 (IP-10) release) and their ability to eliminate cancer cells (via cytotoxic mediators and phagocytosis). Hemin also reduced the mitochondrial membrane potential (MMP) and mitochondrial mass in macrophages. 3,4DHPAA and 4HPAA, by stimulating glycolysis and PPP, prevented the impairment of the macrophage anticancer activity induced by hemin. In conclusion, 3,4HPAA and 4HPAA administration could represent a promising strategy for preventing the reduction of macrophage activation induced by hemin.

## 1. Introduction

According to the latest data provided by the World Health Organization, cancer is the second leading cause of death globally, and is responsible for an estimated 9.6 million deaths in 2018 [1]. Globally, about one in six deaths are due to cancer [2]. Macrophages are innate immune cells that have a pivotal role in cancer prevention, as upon activation, they can identify emerging cancer cells and eliminate them [3]. Macrophages kill cancer cells through phagocytosis and the production of cytotoxic soluble factors, such as cytokines and chemokines [4,5]. Metabolic reprogramming, characterized by high glycolysis and low oxidative phosphorylation (OXPHOS), is critical for macrophage activation towards the M1 phenotype that is characterized by proinflammatory and anticancer properties [6].

**Scheme 1:**
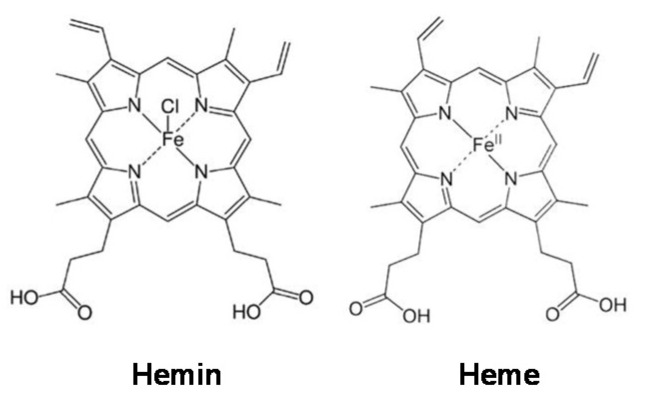
Hemin and heme structures.

In 2009, WHO indicated that almost one-third of cancer-related deaths are associated with the five leading behavioral and dietary risks, namely high body mass index, diet, lack of physical activity, tobacco, and alcohol use. Red meat and processed meat were classified as “probably carcinogenic to humans” and as “carcinogenic to humans”, respectively, by The Agency for Research on Cancer (IARC) in 2015 [7]. In the development of several types of cancer, such as colorectal (CRC), lung, and breast cancers, the role of a high percentage of meat in diets has been implicated [8]. For example, the risk of CRC increases by 17% with a daily intake of 100 g of red meat [9]. Myoglobin (a protein containing a heme group) from red meat can be oxidized into metmyoglobin under acidic conditions in the gastrointestinal tract, causing the dissociation of hemin from metmyoglobin [10,11,12]. Hemin, a covalently modified porphyrin formed from heme, contains a ferric iron (Fe^3+^) ion with a coordinating chloride ligand (Scheme 1) [13]. Hemin has been demonstrated to be involved in CRC initiation, promoting colon carcinogenesis through mechanisms involving oxidative damage [14,15,16]. We recently found that hemin, in addition to promoting malignant transformation in normal colon epithelial cells by inducing intracellular and mitochondrial reactive oxygen species (ROS) production and DNA oxidative damage, can also cause metabolic reprogramming by decreasing MMP and the activity of complexes I and II of the electron transport chain [17]. In addition, hemin has also been shown to reduce MMP in bovine aortic endothelial cells (BAECs), which was associated with a decrease in basal and maximal mitochondrial respiration [18]. Although it is known that metabolic reprogramming in macrophages is critical for their activation and that hemin can induce cellular metabolic changes, the role of hemin in macrophage activation towards an anticancer phenotype has not yet been addressed.

There is a negative correlation between diets containing abundant vegetables, fruits, and grains, and the risk of cancer [19]. The flavonol quercetin (QUE) is the most ubiquitous polyphenol in fruits and vegetables, specifically abundant in apples and onions [20]. Kaempferol, another member of the flavonols, is abundantly found in tea, broccoli, apples, strawberries, and beans [21]. Proanthocyanidins, oligomeric compounds formed from catechin and epicatechin molecules, account for a major fraction of the total flavonoids ingested in Western diets [22]. These polyphenols exert their anticancer effects though mechanisms related to apoptosis, cell cycle arrest, regulation of signaling pathways, and antioxidant response [23,24,25]. Though many healthy systemic effects in humans have been attributed to these flavonoids, they are barely absorbable in the gastrointestinal tract and as a result they accumulate in the lumen [26,27].

Flavonoids can be metabolized by the microbiota in the colon; the metabolite produced can be absorbed and thus exert biological effects in the organism, such as antimicrobial, anti-inflammatory, and antioxidant activities [28]. The major microbial metabolite of QUE and its glycosylated derivatives is 3,4-dihydroxyphenylacetic acid (3,4DHPAA) [29,30,31,32,33,34,35,36], which also has potent antioxidant properties. It has been shown that this metabolite has the highest free radical scavenging activity among several flavonoid metabolites tested in vitro, and that it may reduce plasma lipid peroxidation in vivo [37,38]. Recently, we found that 3,4DHPAA is more effective than QUE in preventing hemin-induced malignant transformation and metabolic reprogramming in normal colon epithelia cells [17]. 4-hydroxyphenylacetic acid (4HPAA) is the major microbiota-derived metabolite of proanthocyanidins and kaempferol [39,40], which possesses antioxidant [41], anxiolytic [42], and antiplatelet properties [43]. These metabolites are detected in human plasma and urine after the ingestion of a standard diet [44] or flavonoid-rich foodstuffs [45,46], as well as after the administration of flavonoid supplements [47]. Although 3,4DHPAA and 4HPPA are abundantly produced in the colon, and can be absorbed, their role in cancer prevention remains unknown.

The present study aimed to determine the effect of hemin on the anticancer properties of macrophages. The role of 3,4DHPAA and 4HPAA in metabolic reprogramming and activation of macrophages that leads to the elimination of cancer cells was also investigated. Based on the capacity of 3,4DHPAA to interfere with cell energy metabolism [17], we postulated that the beneficial effects of 3,4DHPAA and 4HPAA against the macrophage inactivation induced by hemin are related to their ability to stimulate glycolysis and the pentose phosphate pathway (PPP).

## 2. Materials and Methods

### 2.1. Culturing Conditions and Assay Format

**Scheme 2:**
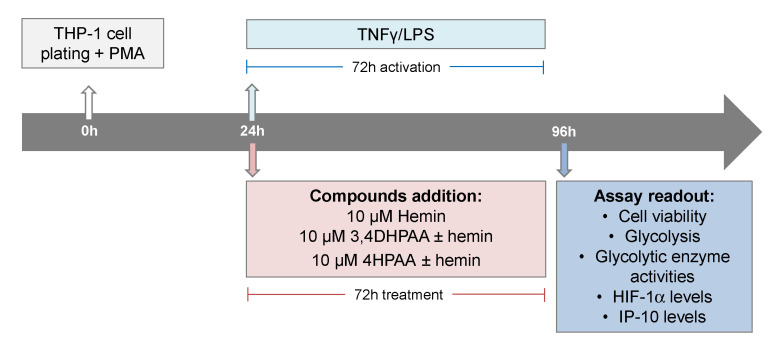
Assay format and compound addition format.

THP-1 (ATCC TIB202) cells, an in vitro model extensively used to study human monocytes and macrophages [48], were maintained in RPMI media (Cat# 11875093, Life Technologies, Carlsbad, CA, USA) supplemented with 10% heat-inactivated fetal bovine serum (FBS, Cat# 10437028, ThermoFisher Scientific, Waltham, MA, USA) at 37 °C in a humidified incubator with 5% CO_2_. The cells were subcultured every 2 to 3 days in order to maintain a cell density between 2 × 10^5^ and 1 × 10^6^ cells/mL. THP-1 cells were seeded in assay plates in RMPI plus 10% FBS medium containing 25 ng of PMA (Cat# P8139, Sigma-Aldrich, St. Louis, MO, USA) per mL in order to induce differentiation of the THP-1 cells. After 24 h of cell plating, macrophages were activated with 5 pg/mL LPS (Cat# L8274, Sigma-Aldrich) and 10 ng/mL INFγ (Cat# SRP3058, Merck, Kenilworth, NJ, USA), and treated with 10 μM hemin (Cat# 51280, Sigma-Aldrich), 10 μM 3,4DHPAA (Cat# 850217, Sigma-Aldrich), or 10 μM 4HPAA (Cat# H50004, Sigma-Aldrich) for 72 h (Scheme 2).

### 2.2. A375 Cells Overexpressing Red Fluorescence Protein

The melanoma cells A375 cells (ATCC^®^ CRL-1619™) were used in this study for the epidemiological relevance of this type of cancer, as melanoma accounts for the majority of skin cancer deaths and its incidence has risen faster than almost any other cancer [49]. A375 cells were infected with purified lentiviral particles for red fluorescence protein and expanded as we previously described [50].

### 2.3. Macrophage Differentiation Biomarkers

THP-1 cells were seeded at 9000 or 2500 cells/well/50 µL in a 384-well plate (CellCarrier Ultra, Cat # 6057300, Perkin Elmer, Baesweiler, Germany) in the absence of PMA. Then, 24 h or 96 h after seeding, cells were fixed in 4% *w*/*v* paraformaldehyde (PFA, Cat# 28908, ThermoFisher) solution for 15 min and washed 3× with PBS (Cat# 14190250, ThermoFisher). In addition, THP-1 cells were seeded at 12,500 cell cells/well/50 µL in a 384-well plate (CellCarrier Ultra), in the presence of 25 ng/mL PMA. After 24, 48, 72, and 96 h, cells were fixed and washed. Fixed cells were blocked for 2 h at room temperature with a buffer containing 2% *w*/*v* BSA and 0.2% *w*/*v* Triton-X 100, then incubated overnight at 4 °C with the primary antibodies (Abs): Anti-CD14 [1H5D8] (1/100, mouse mAb, Cat# ab181470, Abcam, Cambridge, UK) and Anti-CD68 [KP1] (1/100, mouse mAb, Cat# ab955, Abcam) prepared in PBS. After washing 3× with PBS, cells were incubated for 2 h at room temperature with Hoechst 33342 (10 μM, Cat# H1399, Life Technologies) to identify the nucleus; HCS CellMask™ Deep Red Stain (0.2 μg/mL, Cat# H32721, Molecular Probe, Eugene, OR, USA) to identify cytosol and the secondary Ab Alexa Fluor 488 goat anti-mouse IgG (H+L) (1/400, Cat# A11001, ThermoFisher), all prepared in PBS. Cells were washed 3× in PBS and imaged on the Opera Phenix™ High Content Screening System (Perkin Elmer) using a 20× water objective, in which nine fields were imaged per well. Hoechst was detected at 405 nm; Alexa Fluor 488 Ab at 488 nm and HCS CellMask™ Deep Red Stain detected at 655 nm. Images were analyzed using Harmony software (Perkin Elmer) and fluorescence of Alexa Fluor 488 was quantified in the nucleus and cytosol areas.

### 2.4. Cell Viability Inhibition

THP-1 cells were seeded at 12,500 cells/well/50 µL in a 384-well plate (Greiner 384 Plates Black, Cat# 781090, Monroe, NC, USA), in the absence or presence of 25 ng/mL PMA. A375 cells expressing red fluorescence protein were seeded at 2500 cells/well/50 µL in a 384-well plate (Greiner 384 Plates Black). After 24 h, THP-1 and A375 cells were treated with hemin, 3,4DHPAA, and 4HPAA (0.05–50 µM) for 72 h. Cells were incubated for 6 h with 60 µM resazurin (Cat# 14322, Cayman, Ann Arbor, MI, USA) and 10 μM Hoechst 33342 to identify the nucleus. The ability of live cells to reduce resazurin dye was assessed and calculated as we previously described [50].

### 2.5. Immunofluorescence against HIF-1α

Cells were seeded at 12,500 cells/well/50 µL in a 384-well plate (CellCarrier Ultra), in the presence of 25 ng/mL PMA. After 24 h, cells were treated with hemin, 3,4DHPAA, or 4HPAA with or without 5 pg/mL LPS and 10 ng/mL INFγ (Scheme 2). After 72 h, cells were fixed in 4% *w*/*v* PFA solution for 15 min and washed 3× with PBS. Cells were blocked for 2 h, then incubated overnight at 4 °C with anti-HIF-1α (1/200, rabbit pAb, Cat# NB100-134, Novus Biologicals, Littleton, CO, USA) prepared in PBS. After washing 3× with PBS, cells were incubated for 2 h at room temperature with 10 μM Hoechst 33342, 0.2 μg/mL HCS CellMask™ Deep Red Stain, and Alexa Fluor 488 goat anti-rabbit IgG (H+L) (1/400, Cat# A11008, ThermoFisher), all prepared in PBS. Cells were washed 3× in PBS and imaged on the Opera Phenix™ High Content Screening System using a 20× water objective, in which nine fields were imaged per well. Images were analyzed using Harmony software and fluorescence of Alexa Fluor 488 was quantified in the nucleus and cytosol areas [50].

### 2.6. Metabolic Enzyme Activities

The activities of hexokinase, pyruvate kinase, and glucose-6-phosphate dehydrogenase were measured through continuous spectrophotometric assays as previously described [50,51] and specified in Supplementary Materials Table S1.

### 2.7. Extracellular Acidification Rates

Extracellular acidification rates (ECARs) were measured using a Glycolysis kit from Abcam (ab197244). Briefly, cells were seeded at 12,500 cells/well/50 µL in a 384-well plate (Greiner 384 Plates Black), in the presence of 25 ng/mL PMA. After 24 h, cells were treated with hemin, 3,4DHPAA, or 4HPAA with or without 5 pg/mL LPS and 10 ng/mL INFγ (Scheme 2). After 72 h, cells were purged from CO_2_, by incubating cells at 37 °C in a humidified incubator for 2 h prior to the glycolysis assay. The respiratory buffer (containing 1 mM potassium phosphate, 20 mM glucose, 70 mM NaCl, 50 mM KCl, 0.8 mM MgSO_4_, 2.4 mM CaCl_2_) and the Glycolysis Assay Reagent were then added to the cells. Fluorescence at λ excitation 340 nm; λ emission 615 nm was recorded in time at 37 °C, using a Microplate Reader (EnSight, Perkin Elmer). Rates of extracellular acidification are calculated from changes in fluorescence signal over time (120 min). Glycolytic pathway inhibitors, such as 100 mM 2-deoxyglucose (an inhibitor of hexokinase and glucose-6-phosphate isomerase [52], Cat# D8375, Sigma-Aldrich) and 100 mM oxamate (a lactate dehydrogenase (LDH) inhibitor [53], Cat# O2751, Sigma-Aldrich), were added as a control to estimate the glycolysis-dependent ECAR.

### 2.8. IP-10 Levels

Cells were seeded at 50,000 cells/well/200 µL in a 96-well plate (Greiner 96 Plates Clear, Cat# 655180), in the presence of 25 ng/mL PMA. After 24 h, cells were treated with hemin, 3,4DHPAA, or 4HPAA with or without 5 pg/mL LPS and 10 ng/mL INFγ (Scheme 2). After 72 h, media was collected and the IP-10 ELISA kit from Abcam (ab83700) was performed, according to the manufacturer’s instructions. Briefly, samples and IP-10 standards were added to the coated ELISA plate and incubated for 2 h at room temperature. After washing 3× with washing buffer (provided by the kit), the plate was incubated with biotinylated anti-IP-10 for 1 h at room temperature. Wells were washed 3×, and subsequently, they were incubated with Streptavidin-HRP solution for 30 min at room temperature. After washing 3×, plate was incubated with Chromogen TMB substrate for 20 min at room temperature. After adding 100 µL of Stop Reagent (provided by the kit) per well, optical density was measured at 450 nm. Nuclei were stained with 10 μM Hoechst 33,342 for 1 h, imaged on the Opera Phenix™ High Content Screening System at 405 nm (20× water objective), and quantified using Harmony. IP-10 levels were normalized to cell number.

### 2.9. Mitochondrial Parameters

Cells were seeded at 12,500 cells/well/50 µL in a 384-well plate (CellCarrier Ultra), in the presence of 25 ng/mL PMA. After 24 h, cells were treated with hemin, 3,4DHPAA, or 4HPAA with or without 5 pg/mL LPS and 10 ng/mL INFγ (Scheme 2). After 72 h, cells were incubated for 1 h with 800 nM MitoTracker Green (Cat# M7514, ThermoFisher) and 10 μM Hoechst 33342 to evaluate mitochondrial mass; or for 30 min with 250 nM tetramethylrhodamine (TMRE, Cat# T669, ThermoFisher) and 10 μM Hoechst 33342 to evaluate MMP. The fluorescent intensity of Hoechst 33342, MitoTracker Green, and TMRE was detected at 405, 488, and 561 nm, respectively. One µM carbonyl cyanide-4-phenylhydrazone (FCCP, Cat# C2920, Sigma-Aldrich) was used as a control to reduce MMP. Cells were washed 3× in PBS and imaged on the Opera Phenix™ High Content Screening System using a 20× water objective, in which nine fields were imaged per well. Images were analyzed using Harmony software and results were expressed as mean intensity/well.

### 2.10. Cytotoxic Effect of Macrophage

**Scheme 3:**
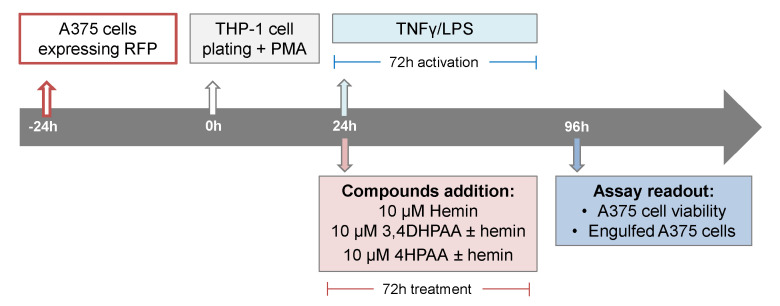
Cytotoxic assays format and compound addition format.

A375 cells expressing red fluorescence protein were seeded at a density of 2000 cells/well/50 µL in a 384-well plate (CellCarrier Ultra). After 24 h, THP-1 cells were seeded at a density of 12,500 cells/well/50 µL in the presence of 25 ng/mL PMA, on top of the cell plate containing the A375 cells. After 24 h, cells were treated with hemin, 3,4DHPAA, or 4HPAA with or without 5 pg/mL LPS and 10 ng/mL INFγ (Scheme 3). After 72 h, cells were incubated for 1 h with Hoechst 33342 and imaged on the Opera Phenix™ High Content Screening System using a 20× water objective, in which nine fields were imaged per well. Nuclei were identified through Hoechst staining at 405 nm and A375 cells at 655 nm. Engulfed A375 cells were identified as disaggregated cells at 655 nm. Images were analyzed using Harmony software.

### 2.11. Statistical Analysis

Data were analyzed by two-way ANOVA followed by Bonferroni’s multiple comparison test, using GraphPad Prism 7 (La Jolla, CA, USA). Unless indicated otherwise, the experiments were performed using three independent culture preparations and in triplicate or quadruplicate. Values are expressed as mean ± SEM. Values with different superscript letters (a, b, c, d, and f) indicate significant differences (*p* < 0.05) between groups. For all bars with the same letter, the difference between the means is not statistically significant.

## 3. Results

### 3.1. Monocyte Differentiation into Macrophages

**Figure 1:**
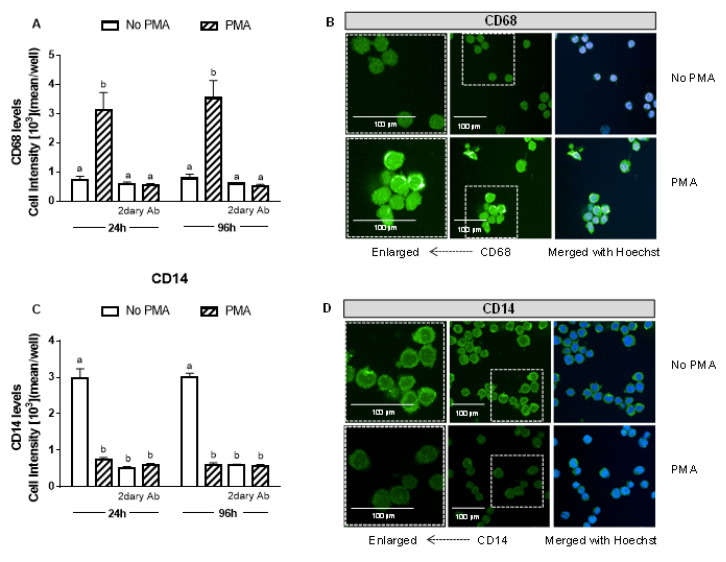
PMA induces monocyte differentiation into macrophages. After 24 and 96 h of differentiation with 25 ng/mL PMA (**A**,**B**), CD68 and (**C**,**D**) CD14 levels were measured and compared to the non-treated THP-1 cells. CD68 and CD14 levels were detected by immunofluorescence using a confocal imaging system (Opera Phenix, Perkin Elmer). Images were acquired with a 20× water objective. Staining intensity levels were analyzed using Harmony software (Perkin Elmer). Nuclei and cytosol were identified through Hoechst (at 405 nm) and CellMask (at 655 nm) staining, respectively. Scale is shown as 100 μm. White dotted frames indicate the section of the image that was enlarged. CD14 (cluster of differentiation 14); Values with different superscript letters (a and b) indicate significant differences (*p* < 0.05) between groups. For all bars with the same letter, the difference between the means is not statistically significant. CD68, cluster of differentiation 68; PMA, phorbol 12-myristate 13-acetate; 2dary Ab, secondary antibody staining.

Human THP-1 monocytes were differentiated into macrophages by incubation with 25 ng of phorbol 12-myristate 13-acetate (PMA)/mL for 24, 48, 72, and 96 h. The presence of the recognized macrophage marker CD68 (cluster of differentiation 68) confirmed the differentiation of monocytes into macrophages [54]. The CD68 signal was already maximal after 24 h incubation with PMA (Figure 1A) and did not increase further at 96 h. The expression of CD14, which decreases with macrophage differentiation [54], was also studied and confirmed the differentiation (Figure 1B). PMA (at the tested concentration and incubation times) was not cytotoxic to macrophages (data not shown).

### 3.2. Hemin Prevented the Increase in IP-10 Release Induced by LPS and INFγ

**Figure 2:**
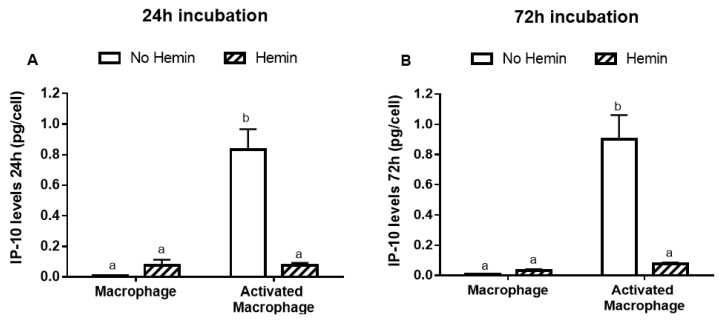
The IP-10 release from macrophages activated with LPS and INFγ is reduced by hemin. Macrophages were incubated for (**A**) 24 or (**B**) 72 h with vehicle in the presence or absence of 10 µM hemin. Macrophages were also activated for (**A**) 24 or (**B**) 72 h with vehicle or 5 pg/mL LPS and 10 ng/mL INFγ in the presence or absence of 10 µM hemin. IP-10 levels in the media were quantified at 450 nm and normalized to the cell number. Values with different superscript letters (a and b) indicate significant differences (*p* < 0.05) between groups. For all bars with the same letter, the difference between the means is not statistically significant. IP-10, interferon-γ-inducible protein 10.

The incubation of macrophages with 5 pg/mL lipopolysaccharide (LPS) and 10 ng/mL interferon gamma (INFγ) for 24 or 72 h activated the macrophages, evidenced by a 100-fold increase in interferon-γ-inducible protein 10 (IP-10) release after 24 or 72 h incubation (Figure 2A,B). Further, 10 µM hemin prevented the increase in IP-10 release induced by LPS and INFγ (Figure 2A,B). Hemin was tested at 2.5, 5, 10, and 20 µM, but only from 10 µM concentration did hemin show an effect in IP-10 release. However, the effect induced by 20 µM was not greater than that observed at 10 µM (Figure S1A). Hemin, LPS, and INFγ were not cytotoxic to macrophages (data not shown). Hemin had no effect in unstimulated macrophages.

### 3.3. Hemin Prevented the Increase in Glycolysis, Metabolic Enzyme Activities, and HIF-1α Levels in Activated Macrophages

**Figure 3:**
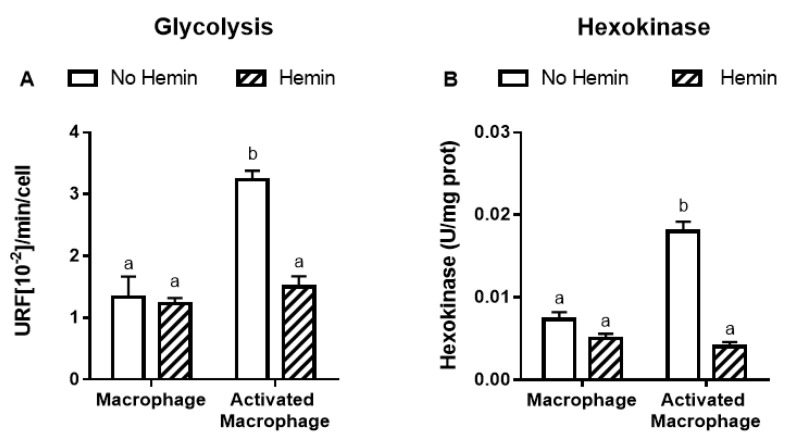

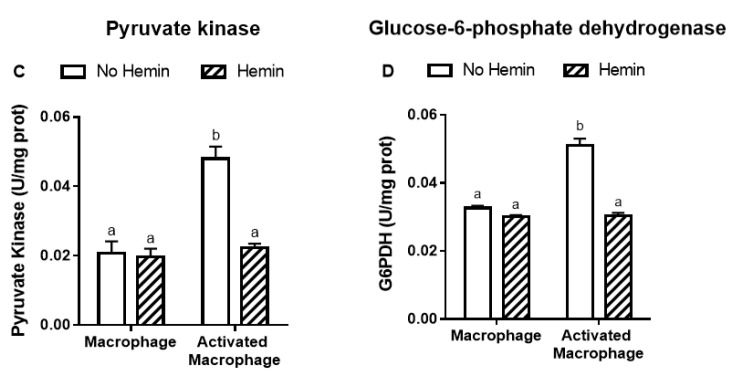
The increased glycolysis and metabolic enzyme activities of activated macrophages are reduced by hemin. (**A**) Glycolysis and the activities of (**B**) hexokinase, (**C**) pyruvate kinase, and (**D**) glucose-6-phosphate dehydrogenase were measured in macrophages and activated macrophages, in the absence or presence of 10 µM hemin for 72 h. Activities of the enzymes were expressed as unit/mg protein, calculated as nmol of NADP reduced/min/mg protein for hexokinase and glucose-6-phosphate dehydrogenase, and as nmol of NADH oxidized/min/mg protein for pyruvate kinase. Values with different superscript letters (a and b) indicate significant differences (*p* < 0.05) between groups. For all bars with the same letter, the difference between the means is not statistically significant. G6PDH, glucose-6-phosphate dehydrogenase.

LPS and INFγ increased the glycolysis by 2.4-fold and the activities of key glycolytic enzymes, hexokinase and pyruvate kinase, by 2.4-fold and 2.3-fold, respectively in macrophages. LPS and INFγ also increased the activity of glucose-6-phosphate dehydrogenase, the enzyme responsible for the first step in the pentose phosphate pathway, by 1.6-fold in macrophages. Hemin prevented the increase in glycolysis and metabolic enzyme activities induced by LPS and INFγ in macrophages (Figure 3).

**Figure 4:**
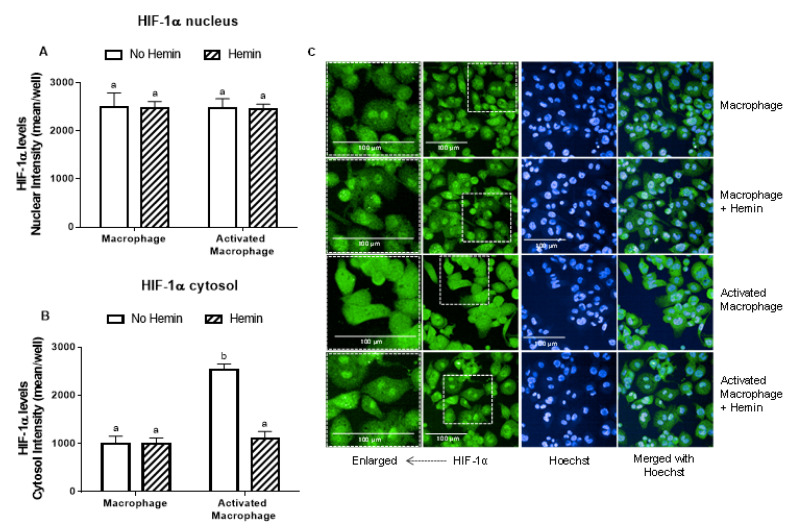
The increase of cytosolic HIF-1α levels of activated macrophages is inhibited by hemin. (**A**) Nuclear and (**B**) cytosolic HIF-1α levels and (**C**) representative images of macrophages and activated macrophages, in the absence or presence of 10 µM hemin for 72 h. HIF-1α levels were detected by immunofluorescence using a confocal imaging system (Opera Phenix, Perkin Elmer). Images were acquired with 20× water objective. Staining intensity levels in the nucleus and cytosolic region were obtained using Harmony software (Perkin Elmer). Nucleus and cytosol were identified through Hoechst (at 405 nm) and CellMask (at 655 nm) staining, respectively. Scale is shown as 100 μm. White dotted frames indicate the section of the image that was enlarged. Values with different superscript letters (a and b) indicate significant differences (*p* < 0.05) between groups. For all bars with the same letter, the difference between the means is not statistically significant. HIF-1α, hypoxia-inducible factor-1 alpha.

LPS and INFγ did not alter the levels of HIF-1α in the nucleus; however, they increased HIF-1α levels in the cytosol by 2.5-fold (Figure 4A,B). This increase observed in activated macrophages was abrogated by hemin (Figure 4B).

### 3.4. Hemin Reduced Mitochondrial Membrane Potential and Mitochondrial Mass of Macrophages

**Figure 5:**
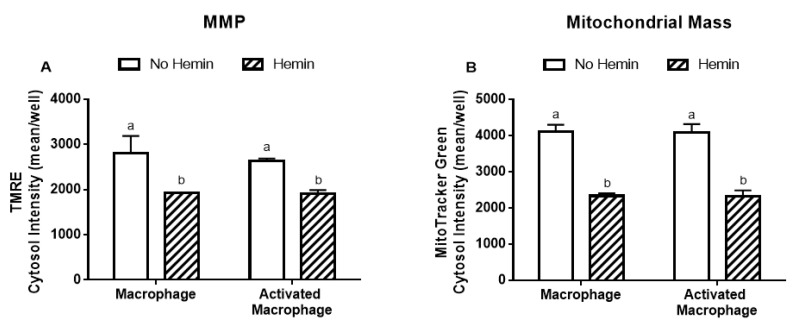
MMP and mitochondrial mass in macrophages are reduced by hemin. (**A**) MMP and (**B**) mitochondrial mass in macrophages and activated macrophages, in the absence or presence of 10 µM hemin for 72 h. MMP and mitochondrial mass were detected by immunofluorescence using a confocal imaging system. Values with different superscript letters (a and b) indicate significant differences (*p* < 0.05) between groups. For all bars with the same letter, the difference between the means is not statistically significant. MMP, mitochondrial membrane potential; TMRE, tetramethylrhodamine.

LPS/INFγ did not alter the MMP and mitochondrial mass in macrophages (Figure 5A,B). Hemin reduced MMP by 32% and 27% in macrophages and in activated macrophages, respectively (Figure 5A). Hemin reduced mitochondrial mass by 43%, regardless of macrophage activation (Figure 5B).

### 3.5. Hemin Prevented the Cytotoxicity of Activated Macrophages and Their Engulfment Capacity

**Figure 6:**
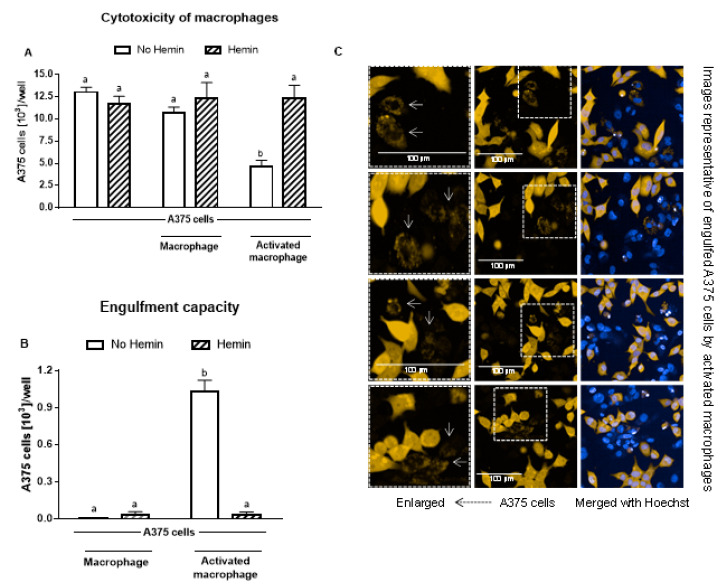
The increased cytotoxic effect and engulfment capacity of activated macrophages is reduced by hemin. (**A**) Cytotoxic effect of macrophages and activated macrophages against A375 cells, in the absence or presence of 10 µM hemin for 72 h. A375 cells were counted using Harmony software (Perkin Elmer), by identifying the cells at 655 nm using a confocal imaging system. (**B**) Engulfment of A375 cells by macrophages and activated macrophages against A375 cells, in the absence or presence of 10 µM hemin for 72 h. (**C**) Engulfed A375 cells by activated macrophages were identified as disrupted cells at 655 nm using a confocal imaging system. Nuclei of macrophages and A375 cells were identified through Hoechst (at 405 nm). Values with different superscript letters (a and b) indicate significant differences (*p* < 0.05) between groups. For all bars with the same letter, the difference between the means is not statistically significant.

Macrophages did not cause a reduction in A375 melanoma cells; however, upon activation, they reduced the A375 cell number by 56% (Figure 6A). In the presence of hemin, the ability of activated macrophages to kill A375 cells was completely abolished (Figure 6A). In addition, hemin inhibited the ability of macrophages to engulf A375 cells (Figure 6B). Hemin did not exert a cytotoxic effect on A375 cells (Figure 6A).

### 3.6. 3,4DHPAA and 4HPAA Increased IP-10 Release in Macrophages and Prevented the Hemin-Induced Reduction of IP-10 Release in Activated Macrophages

**Figure 7:**
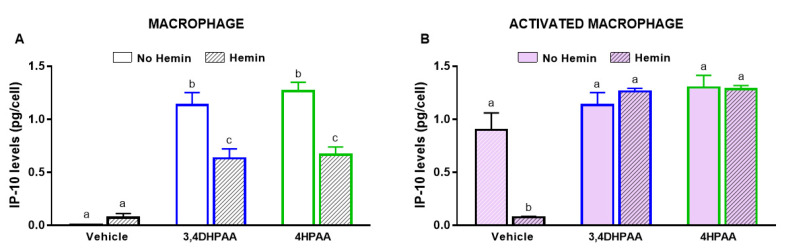
3,4DHPAA and 4HPAA induce IP-10 release in macrophages and hemin reduces IP-10 release in macrophages. Levels of IP-10 in the media of (**A**) macrophages and (**B**) activated macrophages incubated with 10 µM 3,4DHPAA or 10 µM 4HPAA, in the presence or absence of 10 µM hemin for 72 h. Values with different superscript letters (a and b) indicate significant differences (*p* < 0.05) between groups. For all bars with the same letter, the difference between the means is not statistically significant. 3,4DHPAA, 3,4-dihydroxyphenylacetic acid; 4HPAA, 4-hydroxyphenylacetic acid; IP-10, interferon-γ-inducible protein 10.

3,4DHPAA and 4HPAA induced IP-10 release in macrophages by 136-fold and 152-fold, respectively (Figure 7A); however, they did not further increase the IP-10 release of activated macrophages (Figure 7B). In the presence of hemin, the ability of 3,4DHPAA and 4HPAA to induce IP-10 release in macrophages was reduced by 44% and 47%, respectively (Figure 7A). In activated macrophages, 3,4DHPAA and 4HPAA completely prevented the drop in IP-10 release induced by hemin (Figure 7B). The metabolites were also tested at 2.5, 5 and 20 µM, but only from 10 µM concentration were they able to fully prevent the hemin effects (Figure S1B,C). 3,4DHPAA and 4HPAA were not cytotoxic to macrophages (data not shown).

### 3.7. 3,4DHPAA and 4HPAA Increased Glycolysis, Metabolic Enzyme Activities, and HIF-1α Levels in Macrophages and Abrogated Hemin-Induced Alterations in Activated Macrophages

**Figure 8:**
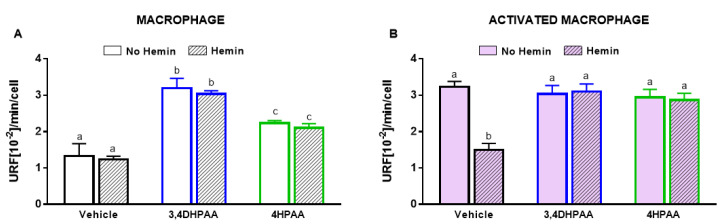
3,4DHPAA and 4HPAA induce glycolysis in macrophages and hemin reduces glycolysis in activated macrophages. Glycolysis of (**A**) macrophages and (**B**) activated macrophages incubated with 10 µM 3,4DHPAA or 10 µM 4HPAA, in the presence or absence of 10 µM hemin for 72 h. Values with different superscript letters (a, b and c) indicate significant differences (*p* < 0.05) between groups. For all bars with the same letter, the difference between the means is not statistically significant. 3,4DHPAA, 3,4-dihydroxyphenylacetic acid; 4HPAA, 4-hydroxyphenylacetic acid.

3,4DHPAA and 4HPAA increased glycolysis in macrophages by 2.4-fold and 1.7-fold, respectively, and regardless of the presence of hemin (Figure 8A). In activated macrophages, 3,4DHPAA and 4HPAA completely prevented the decrease in glycolysis induced by hemin (Figure 8B).

**Figure 9:**
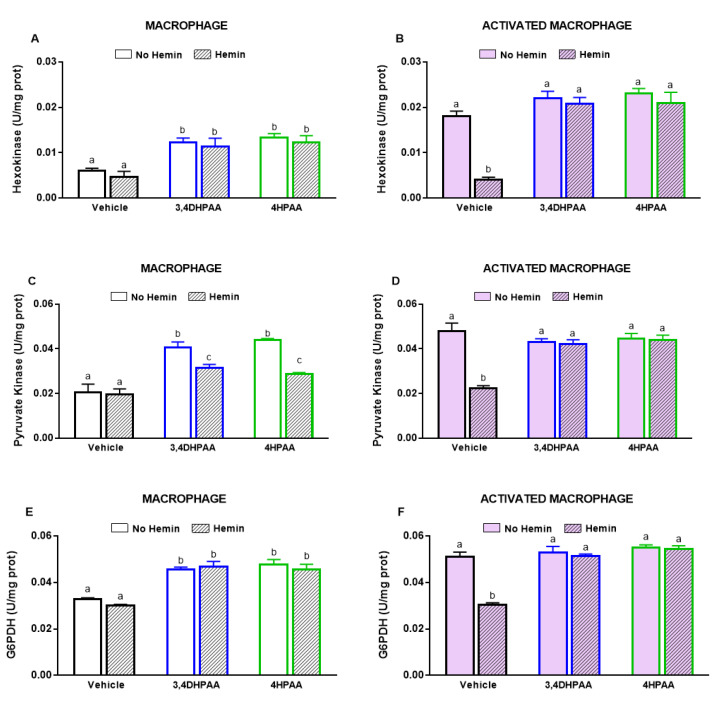
3,4DHPAA and 4HPAA increase metabolic enzyme activities in macrophages and hemin reduces them in activated macrophages. Hexokinase activity in (**A**) macrophages and (**B**) activated macrophages; pyruvate kinase activity in (**C**) macrophages and (**D**) activated macrophages; and glucose-6-phosphate dehydrogenase activity in (**E**) macrophages and (**F**) activated macrophages. Enzyme activities were measured in macrophages incubated with 10 µM 3,4DHPAA or 10 µM 4HPAA, in the presence or absence of 10 µM hemin for 72 h. Values with different superscript letters (a, b and c) indicate significant differences (*p* < 0.05) between groups. For all bars with the same letter, the difference between the means is not statistically significant. 3,4DHPAA, 3,4-dihydroxyphenylacetic acid; 4HPAA, 4-hydroxyphenylacetic acid; G6PDH, glucose-6-phosphate dehydrogenase.

3,4DHPAA increased the activities of hexokinase (Figure 9A) and pyruvate kinase (Figure 9C) by 2-fold and G6PDH activity by 1.4-fold in macrophages (Figure 9E). 4HPAA increased hexokinase, pyruvate kinase, and G6PDH activities in macrophages by 2.2-, 2.1-, and 1.6-fold, respectively (Figure 9A,C,E). Hemin did not alter the effects of both metabolites on the activities of hexokinase and G6PDH; however, it reduced the effect of 3,4DHPAA and 4DPAA on pyruvate kinase activity by 23% and 35%, respectively (Figure 9A,C,E). In activated macrophages, 3,4DHPAA and 4HPAA fully prevented a hemin-mediated reduction of the activities of metabolic enzymes (Figure 9B,D,F).

**Figure 10:**
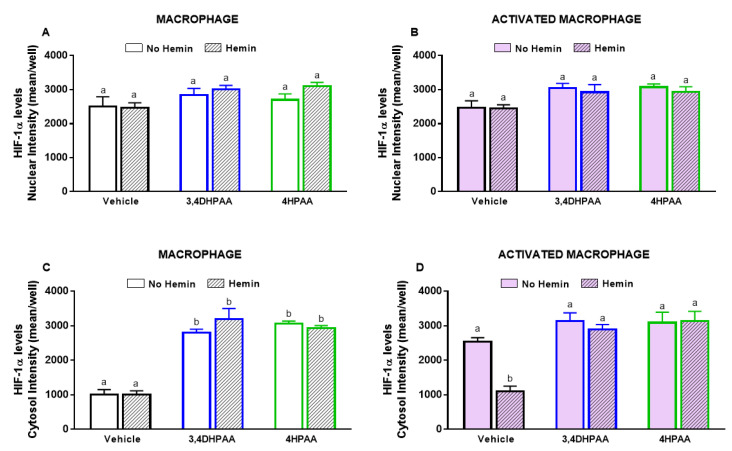
3,4DHPAA and 4HPAA increase cytosolic HIF-1α levels in macrophages and hemin reduces it in activated macrophages. Nuclear HIF-1α levels in (**A**) macrophages and (**B**) activated macrophages; cytosolic HIF-1α levels in macrophages (**C**) and (**D**) activated macrophages. HIF-1α levels were measured in macrophages incubated with 10 µM 3,4DHPAA or 10 µM 4HPAA, in the presence or absence of 10 µM hemin for 72 h. HIF-1α levels were detected by immunofluorescence using a confocal imaging system (Opera Phenix, Perkin Elmer) and quantified using Harmony software (Perkin Elmer). Values with different superscript letters (a and b) indicate significant differences (*p* < 0.05) between groups. For all bars with the same letter, the difference between the means is not statistically significant. 3,4DHPAA, 3,4-dihydroxyphenylacetic acid; 4HPAA, 4-hydroxyphenylacetic acid; HIF-1α, hypoxia-inducible factor-1 alpha.

3,4DHPAA, 4HPAA, and hemin did not alter the nuclear levels of HIF-1α in both macrophages (Figure 10A) and activated macrophages (Figure 10B). 3,4DHPAA and 4HPAA increased cytosol levels of HIF-1α in macrophages by 2.8-fold and 3.1-fold, respectively (Figure 10C). Hemin did not alter the effect of the metabolites in macrophages (Figure 10C). In activated macrophages, 3,4DHPAA and 4HPAA completely prevented the decrease in cytosolic HIF-1α levels induced by hemin (Figure 10C).

### 3.8. 3,4DHPAA and 4HPAA Increased the Ability of Macrophages to Kill A375 Cells and Prevented the Inhibitory Effect of Hemin on Cancer Cell Elimination Capacity in Activated Macrophages

**Figure 11:**
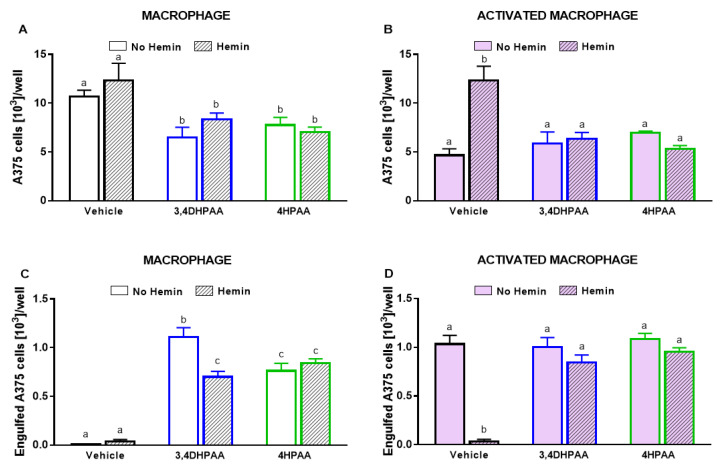
3,4DHPAA and 4HPAA increased the ability of macrophages to kill cancer cells and hemin reduces this ability in activated macrophages. Cytotoxic effect of (**A**) macrophages and (**B**) activated macrophages against A375 cells, incubated with 10 µM 3,4DHPAA or 10 µM 4HPAA, in the presence or absence of 10 µM hemin for 72 h. A375 cells were counted using Harmony software, by identifying the cells at 655 nm using a confocal imaging system. Engulfment of A375 cells by (**C**) macrophages and (**D**) activated macrophages, incubated with 10 µM 3,4DHPAA or 10 µM 4HPAA, in the presence or absence of 10 µM hemin for 72 h. Engulfed A375 cells were identified as disrupted cells at 655 nm using a confocal imaging system. Values with different superscript letters (a and b) indicate significant differences (*p* < 0.05) between groups. For all bars with the same letter, the difference between the means is not statistically significant. 3,4DHPAA, 3,4-dihydroxyphenylacetic acid; 4HPAA, 4-hydroxyphenylacetic acid.

Untreated macrophages did not reduce A375 cell number; however, in the presence of LPS/IFNγ, 3,4DHPAA, or 4HPAA, macrophages were able to reduce A375 cell numbers (Figure 11A,B). The metabolites did not exert direct cytotoxic effects on A375 cells (data not shown). 3,4DHPAA and 4HPAA induced a reduction of the A375 cell number when co-cultured with macrophages by 39% and 27%, respectively (Figure 11A). This effect of the metabolites was not altered by the presence of hemin (Figure 11A). The ability of hemin to reduce the cytotoxicity of activated macrophages on A375 cells was completely prevented by 3,4DHPAA and 4HPAA (Figure 11B). Hemin has no cytotoxic effect in A375 cells (Figure 6A).

Consistently, macrophages engulfed A375 cells only upon activation with LPS/IFNγ, 3,4DHPAA, or 4HPAA (Figure 11C,D). 3,4DHPAA and 4HPAA increased the ability of macrophages to engulf A375 cells (Figure 11C). Hemin did not interfere with the ability of 4HPAA to increase the engulfment capacity of macrophages (Figure 11C), whereas it reduced this effect of 3,4DHPAA by 37% (Figure 11C). In addition, hemin inhibited the ability of activated macrophages to engulf A375 cells, which was completely prevented by 3,4DHPAA and 4HPAA (Figure 11D).

### 3.9. 3,4DHPAA and 4HPAA Did Not Alter MMP and Mitochondrial Mass of Macrophages

3,4DHPAA and 4HPAA did not result in alterations in MMP and mitochondrial mass of macrophages (Supplementary Material Figure S2A,C) and activated macrophages (Supplementary Materials Figure S2B,D). 3,4DHPAA and 4HPAA did not prevent the hemin-induced decrease in MMP and mitochondrial mass of macrophages and activated macrophages (Supplementary Materials Figure S2A–D).

## 4. Discussion

### 4.1. Hemin Prevented the Increase in IP-10 Release Induced by LPS and INFγ: Role of 3,4DHPAA and 4HPAA

Activated macrophages with proinflammatory and antitumor phenotypes, known as the M1 macrophages, can be induced by LPS and IFNγ [55]. We observed that hemin prevented macrophage activation, evidenced by the inhibition of IP-10 release induced by LPS and INFγ (Figure 2A,B). Consistently, it has been shown that hemin dose-dependently decreases proinflammatory cytokines interleukin (IL)-1β and tumor necrosis factor (TNF)-α levels induced by oxidized low-density lipoprotein (ox-LDL) in a macrophage-derived foam cell model from U937 cell line [56]. In addition, we have shown that 10 µM hemin increases ROS production and promotes DNA oxidative damage in cancer and normal colon cells [17]. Consistently, it has been reported that hemin (25 µM) induces protein oxidation and lipid peroxidation in BAECs [18]. Moreover, we found that 3,4DHPAA was able to prevent the hemin-induced oxidative damage in cancer and normal colon cells [17] and here we found that the metabolites induced the release of the proinflammatory cytokine in macrophages regardless of the presence of hemin (Figure 7A). These findings suggest that the mechanism underlying the ability of the metabolites to prevent hemin-induced macrophage inactivation may rely on their antioxidant and proinflammatory properties. However, more studies are needed to address the effect of hemin and metabolites in the macrophages pro- and antioxidant systems.

### 4.2. Hemin Prevented the Increase of Glycolysis and Metabolic Enzyme Activities, and the Stabilization of HIF-1α in Activated Macrophages: Effect of 3,4DHPAA and 4HPAA

Activated M1-macrophages are characterized by high glycolysis, and low OXPHOS [57]. Our results showed that LPS and IFNγ, as well as the metabolites, 3,4DHPAA and 4HPAA, increased glycolysis (Figure 3A and Figure 8A,B), and the activities of the glycolytic and PPP enzymes (Figure 3B,D and Figure 9A–F), which is in line with the metabolic adaptations that macrophages undergo upon activation towards proinflammatory and anticancer phenotypes [58,59,60]. Interestingly, downregulation of the glycolytic enzyme, hexokinase, inhibits inflammasomes in macrophages upon LPS activation [60]. Inflammasomes are multi-protein complexes involved in the maturation and secretion of proinflammatory cytokines of macrophages [61]. In addition, genetic deletion or pharmacological inhibition of another key glycolytic enzyme, pyruvate kinase, has been shown to prevent macrophage activation towards the M1 phenotype in response to LPS and IFN-γ [59]. In the same line, when macrophage glucose-6-phosphate dehydrogenase, a PPP enzyme, is chemically inhibited or genetically suppressed, LPS-induced proinflammatory gene expression is attenuated [58]. Considering the pivotal role that these metabolic enzymes have in macrophage activation, it is possible that 3,4DHPAA and 4HPAA promote IP-10 release in macrophages by increasing the activities of hexokinase, pyruvate kinase, and glucose-6-phosphate dehydrogenase (Figure 9A–F). Moreover, the notion that hemin prevents LPS/ IFNγ-induced macrophage activation by inhibiting glycolysis is supported by the fact that 2-deoxyglucose has been shown to decrease the activation of macrophages towards proinflammatory and anticancer phenotypes (Supplementary Materials Figure S3) [62,63]. However, the role of hemin on macrophages acyloxyacyl hydrolase or lipoprotein lipase activities, as an additional mechanism for hemin to affect macrophage response via LPS inactivation, still remains to be explored.

HIF-1α is an essential transcription factor that mediates energy metabolism under hypoxic conditions [64]. It has been shown to increase glycolysis by upregulating the transcription of glycolytic enzymes, such as hexokinase and pyruvate kinase [65,66]. HIF-1α has been reported to also increase PPP by upregulating the transcription of glucose-6-phosphate dehydrogenase [67]. Here, we showed that LPS and IFNγ, as well as the metabolites, 3,4DHPAA and 4HPAA, stabilized HIF-1α in macrophages under normoxia, as evidenced by increased protein levels in the cytosol. Hemin, however, decreased HIF-1α stabilization induced by proinflammatory signals and the metabolites (Figure 4A,B, Figure 10A–D). These findings suggest that 3,4DHPAA and 4HPAA, by preventing hemin-induced destabilization of HIF-1α, promote the expression of glycolytic enzymes, which could contribute to the increase in glycolysis and macrophage activation.

### 4.3. Hemin Decreased the Cytotoxic Effect and Engulfment Capacity of Activated Macrophages: Effect of 3,4DHPAA and 4HPAA

Inhibition of glycolysis affects many functions of macrophages with proinflammatory and anticancer phenotypes, such as phagocytosis and the secretion of proinflammatory cytokines, events that ultimately contribute to the elimination of cancer cells [68,69,70,71]. Overexpression of the glucose transporter, GLUT1, in RAW264.7 macrophages resulted in an elevated secretion of inflammatory mediators, increased glucose uptake and metabolism, enhanced PPP intermediates, and decreased oxygen consumption rate [68]. All these events were attenuated by the pharmacological inhibition of glycolysis, using 2-deoxyglucose, highlighting the pivotal role of this metabolic pathway in the proinflammatory cytotoxic effect of macrophages [68]. Consistently, it has been found that 2-deoxyglucose inhibits the phagocytosis of immunoglobulin G, or complement-coated sheep erythrocytes by macrophages [69,70]. Here, we found that hemin and 2-deoxyglucose decreased the cytotoxic effect of activated macrophages against cancer cells (Figure 6 and Supplementary Materials Figure S4). In addition, 2-deoxyglucose suppresses glycolysis and reduces phagocytosis in elicited macrophages (with Brewer thioglycollate broth injection) [71]. We suggest, therefore, that the mechanism underlying the protective effect of 3,4DHPAA and 4HPAA against the hemin-induced decrease of the cytotoxic effect and engulfment capacity of activated macrophages may rely on their ability to increase glycolysis (Figure 11A–D). However, as etomoxir, an inhibitor of fatty acid oxidation, can also decrease the engulfment of tumor cells by macrophages [72], more studies are necessary to evaluate the role of 3,4DHPAA and 4HPAA in fatty acid oxidation and its contribution in metabolite-mediated macrophage activation.

### 4.4. Hemin Decreased MMP and Mitochondrial Mass in Macrophages: Effect of 3,4DHPAA and 4HPAA

A low level of OXPHOS is one of the metabolic features that characterizes activated M1-macrophages [57]. In fact, oligomycin, a compound that inhibits OXPHOS by blocking the F0 subunit of the H^+^-ATPase, has been shown to increase glycolysis and phagocytosis in elicited macrophages [71]. Inhibition of glycolysis with 2-deoxyglucose shifts the glycolytic metabolism of lymphoma cells to a predominantly OXPHOS-dependent metabolism [73] and increases MMP in breast cancer MCF7 cells [74]. However, we found that hemin, despite being able to decrease glycolysis (Figure 3A), failed to increase MMP in macrophages and on the contrary, hemin decreased MMP and mitochondrial mass (Figure 5 A,B). Hemin has also been shown to reduce MMP in BAECs, which was associated with a decrease in the basal and maximal mitochondrial respiration [18]. The MMP generated by proton pumps from complexes I, III, and IV into the intermembrane space of mitochondria is an essential process for energy production during the OXPHOS. Together with the proton gradient, MMP forms the transmembrane potential of hydrogen ions, which is critical to make ATP; in consequence, a drop in MMP compromises OXPHOS [75]. We suggest that hemin impairs the energy metabolism of macrophages, not only by decreasing glycolysis but also by affecting mitochondrial membrane potential, which consequently could alter OXPHOS. 3,4DHPAA and 4HPAA were not able to prevent these mitochondrial impairments induced by hemin, which indicate that their protective effects on macrophage activation may rely mainly on mechanisms involving the glycolytic pathway. However, to evaluate the physiological effect of hemin and the metabolites on macrophage metabolism and mitochondrial function, studies using in vivo models are required

### 4.5. Physiological Relevance of This Study and Limitations

After an intake of only 100 mg of beef, approximately 6.7–30.1 µM hemin could be produced in the colon lumen [76,77,78]. However, considering that most heme is broken down in the enterocyte, and only a small fraction of heme or hemin could be transported intact across the basolateral membrane, the concentration used in this study is unlikely to be achieved in vivo [79,80,81]. The impact of high amounts and frequency of red meat intake on heme and hemin absorption need to be determined. With respect to the limitations of our findings, it is unlikely that the effective concentrations of 3,4DHPAA and 4HPAA used in this study can be reached in vivo. Concentrations of 6.98 and 18.54 µM of 3,4DHPAA and 4HPAA, respectively, have been detected in fecal water after the administration of a standard diet [82]. In addition to the dietary intake of these metabolites’ precursors, the effective concentrations of 3,4DHPAA and 4HPAA that can be reached in plasma will depend on differences in the host microbiota, absorption mechanism, intermediate metabolism, and excretion [83]. Plasma concentrations of 0.05 µM 3,4DHPAA have been found in healthy children [84] and 0.4 µM 4HPAA has been observed in the plasma of healthy adults [85]. A strategy to increase the plasma concentration of these metabolites could be to consume supplements containing their precursors, as well as diets high in flavonoids, especially foods abundant in QUE, proanthocyanidins, and kaempferol. For instance, it has been shown that the urinary excretion of 3,4DHPAA as well as other phenolic acid metabolites doubled in human subjects after consumption of chocolate [86]. Urinary excretion of 4HPAA increased by 40% after the intake of green tea [46]. However, further studies in in vivo models are required to investigate the beneficial role of 3,4HDPAA and 4HPAA in macrophage activation and its role in cancer prevention.

## 5. Conclusions

In conclusion, this study shows that hemin promotes inhibition of glycolysis, glycolytic, and PPP enzyme activities and HIF-1α stabilization, which interfere with macrophage activation and their ability to eliminate cancer cells. 3,4DHPAA, a microbial metabolite of QUE, and 4HPAA, a microbial metabolite of proanthocyanidins and kaempferol, prevented hemin-induced impairment of anticancer activity in macrophages by stimulating glycolysis and PPP. 3,4DHPAA and/or 4HPAA administration could represent a promising strategy to enhance the ability of macrophages to eliminate cancer cells and to prevent the detrimental effects of hemin on macrophages. However, additional experiments are required to evaluate the protective effects of 3,4DHPAA and 4HPAA in in vivo models under dietary interventions, such as a high-meat diet.

## Supplementary Materials

Supplementary materials can be found at https://www.mdpi.com/2076-3921/9/11/1109/s1.
