# Supplementary material for: Hemin Prevents Increased Glycolysis in Macrophages upon Activation: Protection by Microbiota-Derived Metabolites of Polyphenols
Source: Antioxidants (Basel). 2020 Nov 11;9(11):1109. doi: 10.3390/antiox9111109 (PMC7696608; doi:10.3390/antiox9111109)
Supplement: Supplementary file 1 [file antioxidants-09-01109-s001.pdf]

Supplementary Table 1. Kinetic assay to evaluate the activity of metabolic enzymes.

ENZYME	REACTION MIX (FINAL CONCENTRATIONS)	INITIATING SUBSTRATE	READOUT
**HEXOKINASE**	60 mM Triethanolamine (pH 7.4), 3.6 mM ATP, 1 mM β-NADP, 8.5 mM $MgCl_2$, 5U/mL Glucose 6-phosphate dehydrogenase (Sigma G8404)	250 mM D-glucose	Reduction of NADP at 340 nm
**PYRUVATE KINASE**	1x PBS (1 mM KH_2PO_4, 155 mM NaCl, 3 mM Na_2HPO_4, pH 7.4), 5 mM ATP, 40 mM $MgCl_2$, 50 mM KCl, 0.6 mM β-NADH, 0.5U/mL Lactate dehydrogenase (Sigma L2500)	20 mM phosphoenolpyruvate	Oxidation of NADH at 340 nm
**GLUCOSE-6-PHOSPHATE DEHYDROGENASE**	60 mM Triethanolamine (pH 7.4), 8.5 mM $MgCl_2$, 2 mM β-NADP	15 mM glucose6-phosphate	Reduction of NADP at 340 nm

Supplementary Figure 1

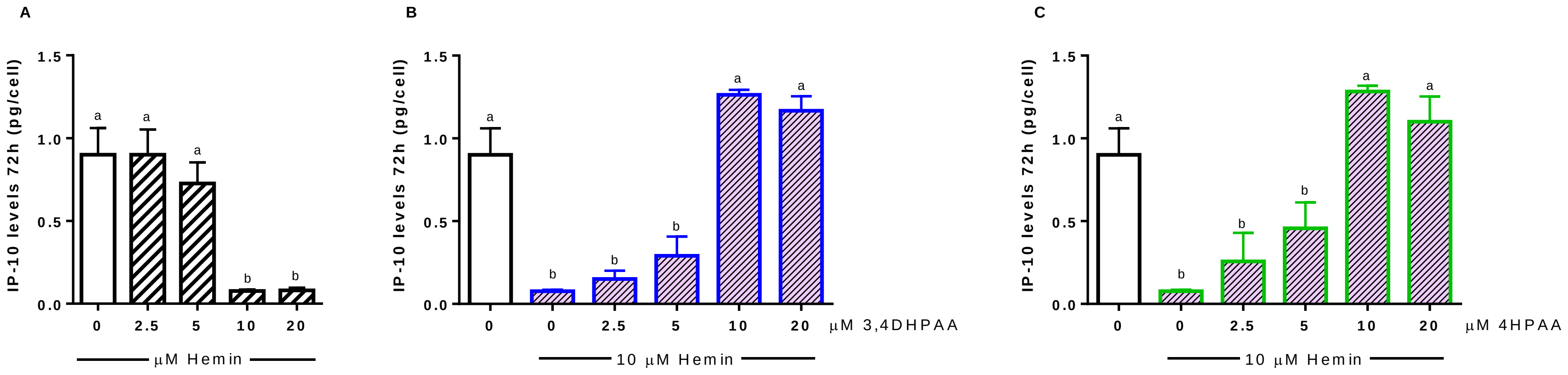

Supplementary Figure 1. 3,4DHPAA and 4HPAA prevent the hemin-induced reduction of IP-10 release in activated macrophages. Levels of IP-10 in the media of activated macrophages after 72h-treatment with: (A) hemin (0 - 20 µM), (B) 3,4DHPAA (0 - 20 µM) in the presence of 10 µM hemin and (C) 4HPAA (0 - 20 µM) in the presence of 10 µM hemin. Values are expressed as mean ± SEM, from three independent culture preparations, each treatment performed in triplicate. For all bars with the same letter, the difference between the means is not statistically significant. Values with different letters indicate significant differences ($p < 0.05$) between bars (one-way ANOVA, Bonferroni post-test). 3,4DHPAA, 3,4-dihydroxyphenylacetic acid; 4HPAA, 4-hydrohyphenylaceic acid; IP-10, interferon-γ-inducible protein 10.

Supplementary Figure 2

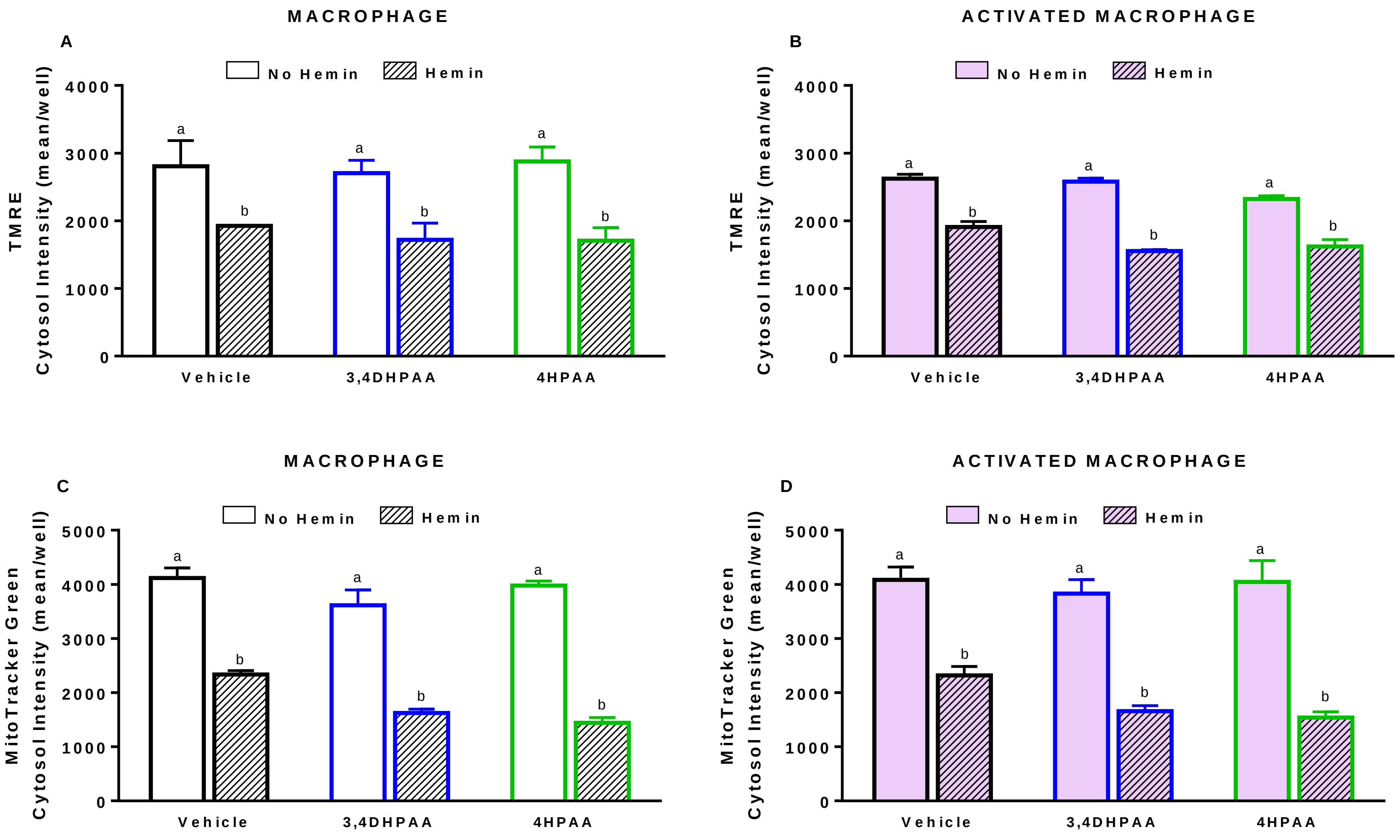

Supplementary Figure 2. 3,4DHPAA and 4HPAA prevent the decrease of MMP and mitochondrial mass induced by hemin in macrophages and activated macrophages. MMP of (A) macrophages and (B) activated macrophages; and mitochondrial mass of (C) macrophages and (D) activated macrophages, incubated with 10 µM 3,4DHPAA or 10 µM 4HPAA, in the presence or absence of 10 µM hemin for 72h. MMP and mitochondrial mass were detected by immunofluorescence using confocal imaging system. Values are expressed as mean ± SEM, from three independent culture preparations, each treatment performed in quadruplicate. For all bars with the same letter, the difference between the means is not statistically significant. Values with different letters indicate significant differences ($p < 0.05$) between bars (two-way ANOVA, Bonferroni post-test). 3,4DHPAA, 3,4-dihydroxyphenylacetic acid; 4HPAA, 4-hydrohyphenylaceic acid; MMP, mitochondrial membrane potential; TMRE, tetramethylrhodamine.

Supplementary Figure 3

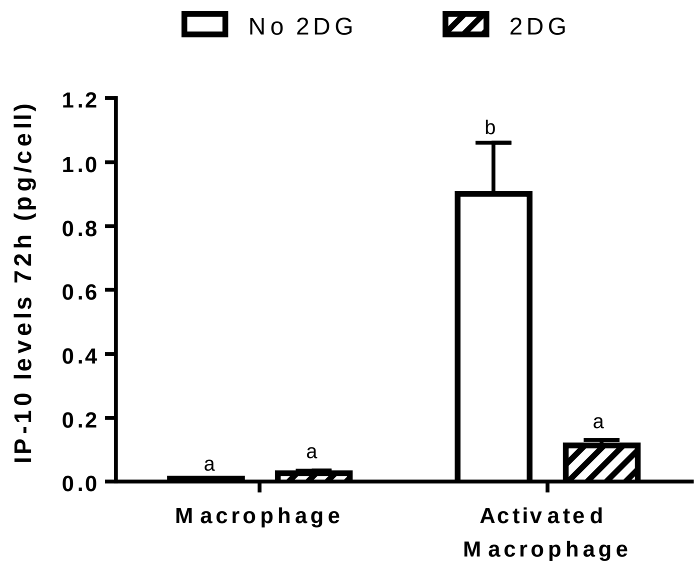

Supplementary Figure 3. 2-deoxyglucose reduces the ability of activated macrophages to release IP-10. Macrophages, were incubated with vehicle in the presence or absence of 100 µM 2-deoxyglucose for 72h. Activated macrophages were incubated with 5 pg/ml LPS and 10 ng/ml INFγ, in the presence or absence of 100 µM 2-deoxyglucose for 72h. IP-10 levels in the media were quantified at 450 nm and normalized to the cell number. Values are expressed as mean ± SEM, from three independent culture preparations, each treatment performed in triplicate. For all bars with the same letter, the difference between the means is not statistically significant. Values with different letters indicate significant differences ($p < 0.05$) between bars (two-way ANOVA, Bonferroni post-test). 2DG, 2-deoxyglucose.

Supplementary Figure 4

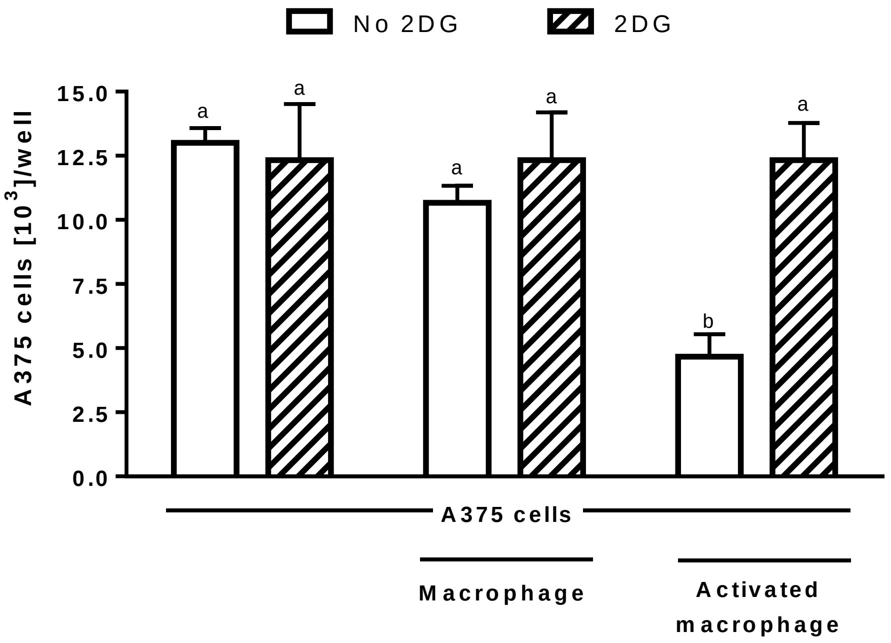

Supplementary Figure 4. 2-deoxyglucose reduces the ability of activated macrophages to kill cancer cells. Macrophages, were incubated with vehicle in the presence or absence of 100 µM 2-deoxyglucose for 72h. Activated macrophages were incubated with 5 pg/ml LPS and 10 ng/ml INFγ, 100 µM 2-deoxyglucose for 72h. A375 cells were counted using Harmony software, by identifying the cells at 655 nm using confocal imaging system. Values are expressed as mean ± SEM, from three independent culture preparations, each treatment performed in triplicate. For all bars with the same letter, the difference between the means is not statistically significant. Values with different letters indicate significant differences ($p < 0.05$) between bars (two-way ANOVA, Bonferroni post-test). 2DG, 2-deoxyglucose.
